# Multidisciplinary Management Considerations in Invasive Lobular Breast Cancer: It Isn’t Just Ductal

**DOI:** 10.1245/s10434-026-20368-y

**Published:** 2026-08-17

**Authors:** Anna C. Beck, Evelyn F. Carroll, Rita A. Mukhtar, Michael R. Cassidy, Meghan R. Flanagan, Lauren A. Gamble, Lillian M. Erdahl

**Affiliations:** 1 Department of Surgery, University of Wisconsin-Madison, Madison, WI USA; 2 Department of Radiology, Mayo Clinic, Rochester, MN USA; 3 Department of Surgery, University of California San Francisco, San Francisco, CA USA; 4 Section of Surgical Oncology, Boston Medical Center, Boston University, Boston, MA USA; 5 Department of Surgery, University of Washington, Seattle, WA USA; 6 Department of Surgery, University of Colorado, Aurora, CO USA; 7 Department of Surgery, University of Iowa, Iowa City, IA USA

## Abstract

**Background:**

Invasive lobular carcinoma (ILC) is the second most common histologic subtype of breast cancer and presents histologically with a unique pattern of discohesive growth. An expert consensus panel was convened at the annual meeting of the American Society of Breast Surgeons to discuss disease specific management considerations and best practice when caring for women with ILC.

**Methods:**

At the annual meeting of the American Society of Breast Surgeons held in Las Vegas, Nevada from April 30 to May 4, 2025, a panel convened to specifically address the intricacies of managing ILC. Panelists completed an independent literature review for each identified subtopic that was summarized and reviewed by panel moderators.

**Results:**

The multidisciplinary management of ILC is nuanced and special considerations are essential to optimize treatment. Magnetic resonance imaging of the breast can improve the accuracy of preoperative staging. Surgeons should account for the limitations of imaging with respect to margin resection in lumpectomy. When considering the application of trials guiding omission of ALND in surgery for ILC, surgeons can rely on several studies that have included subanalyses of patients with ILC confirming safety in appropriately selected patients. Use of neoadjuvant therapy should be based on molecular subtype rather than tumor histology. Genetic testing for patients with ILC should include analysis of *CDH1* mutations.

**Discussion:**

Awareness and consideration of nuances around the management of ILC, including those discussed in the present study, can help clinicians provide optimal, evidenced based care to women presenting with ILC.

Invasive lobular carcinoma (ILC) is the second most common histological subtype of breast cancer, representing 10% of all breast cancers with a projected 33,600 women expected to be diagnosed with ILC in the United States in 2025.^1^ From 2012 to 2021, the incidence of ILC has increased at a higher rate (2.8% per year) compared with other breast cancer subtypes (0.8% per year).^1^ Histologically, ILC has a unique pattern of discohesive growth that necessitates special considerations in evaluation and treatment. A recent report supported by the American Cancer Society utilizing a nationally representative cancer registry from the National Cancer Institute and Centers for Disease Control and Prevention found that ILC is more likely to be diagnosed in older women, be hormone receptor (HR)-positive and HER2-negative, and was twice as likely compared to ductal histology to be larger than 5 cm at the time of diagnosis.^1^ In an effort to provide guidance to breast surgeons on the evaluation and management of this unique cancer type, a panel of experts was convened at the annual meeting of the American Society of Breast Surgeons in May 2025 to address several aspects of care: imaging evaluation, surgical management of the breast, surgical management of the axilla, neoadjuvant systemic therapy, and genetic testing considerations.

## Methods

At the annual meeting of the American Society of Breast Surgeons held in Las Vegas, Nevada from April 30 to May 4, 2025, a panel convened to specifically address the intricacies of managing ILC. A multidisciplinary panel of experts in breast oncology and specifically ILC was organized by the program leadership. The specific subjects the panel sought to address in regard to the management of patients diagnosed with ILC of the breast included imaging, surgical management of the breast, surgical management of the axilla, use of neoadjuvant therapy, and genetics. Panelists completed an independent literature review for each identified topic. The panel and subsequent discussion was moderated by authors ACB and LME. Following the annual meeting, all panelists summarized their literature review and presentation from the annual meeting for inclusion in the present manuscript. All summarized data and literature review was independently reviewed by authors ACB and LME.

## Results

### Imaging in ILC

The histologic features of ILC underlie its imaging appearance, with the hallmark morphologic finding being a single-file growth pattern with discohesive monomorphic tumor cells. This results from the characteristic loss of E-cadherin observed in most ILC cases. Notably, E-cadherin may be detectable on immunohistochemistry (IHC) in up to 23% of cases, but additional staining for p-120 can reveal E-cadherin dysfunction, often due to truncating alterations in its gene, *CDH1*. Because ILC spreads in an infiltrative pattern, it creates little desmoplastic response, consequently creating less clinically apparent palpable mass formation. Clinically, ILC may present as a mass, vague area of palpable thickening, “shrinking breast,” or nipple retraction. Imaging is particularly important in the detection and management of ILC given its often subtle clinical presentation. Although current imaging modalities are not specific for differentiating ILC from other invasive breast cancers, some data suggest that ILC is more likely to present with larger tumors as well as multifocal or contralateral disease.^2,3^ Understanding the imaging features of ILC is critical for accurate diagnosis, early detection, and effective treatment.

**Fig. 1:**
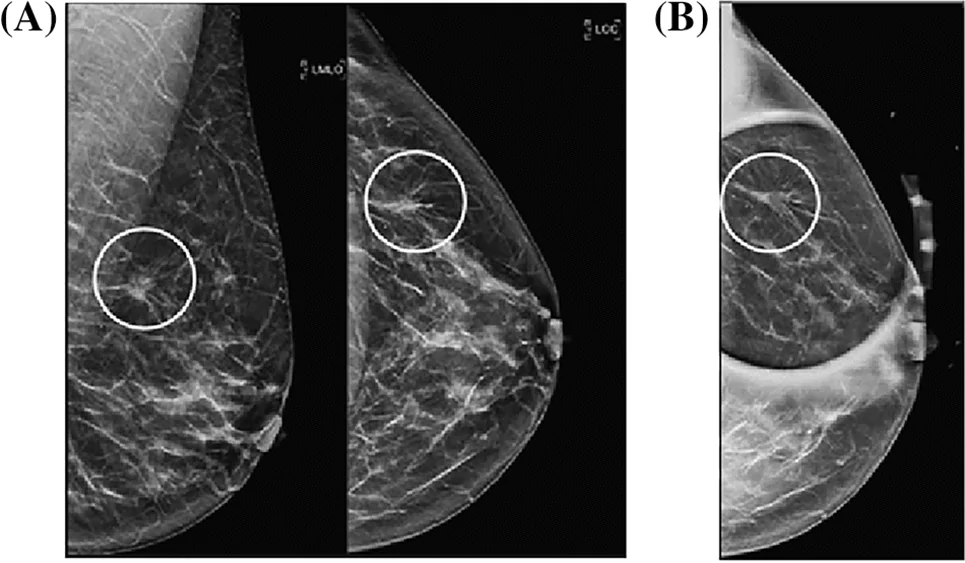
A 55 year-old female presenting for screening mammogram. **a** Left craniocaudal (CC) and mediolateral oblique (MLO) views demonstrate a focal asymmetry in the outer upper left breast at posterior depth. **b** Spot compression CC view shows a persistent spiculated mass

**Fig. 2:**
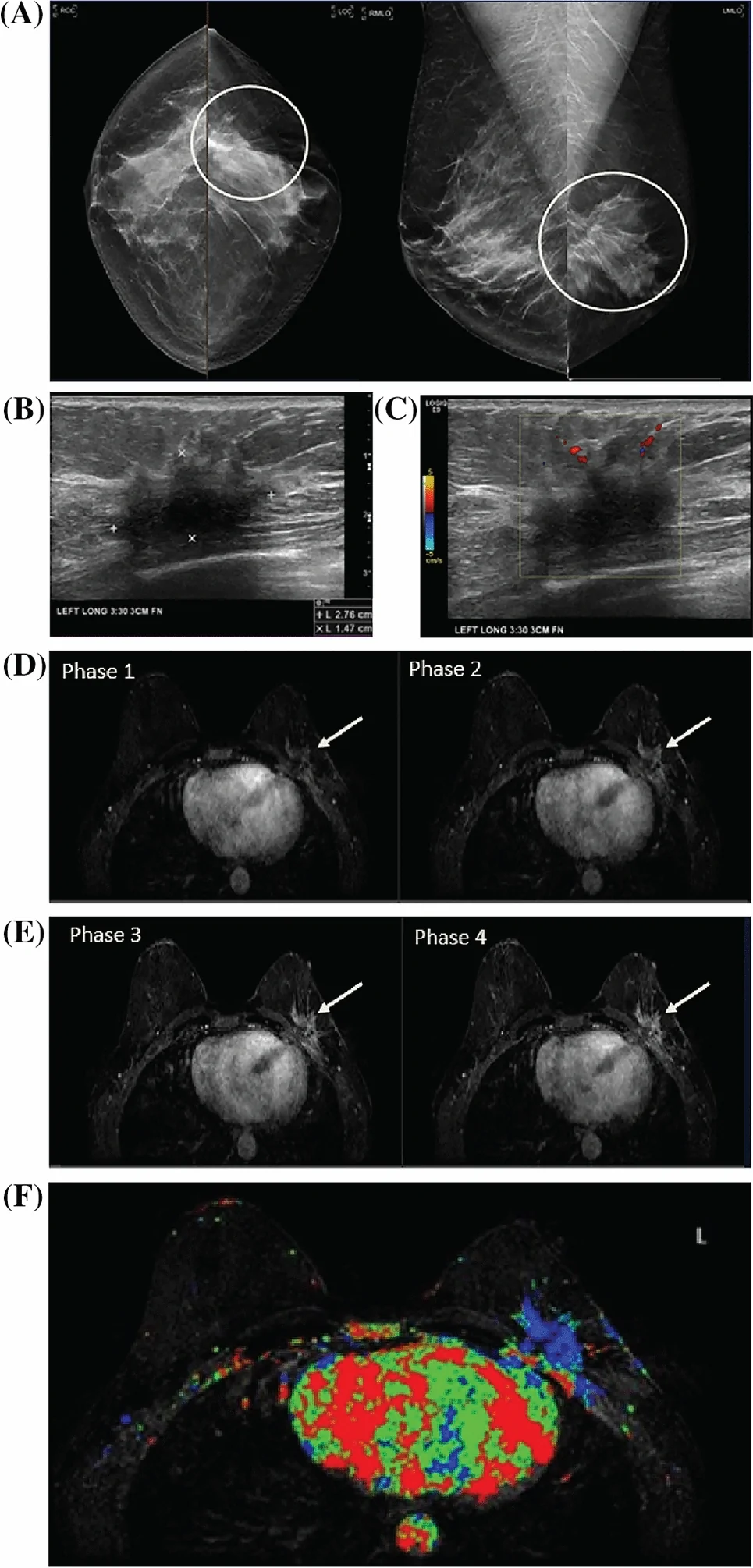
A 56 year-old female presenting for annual screening mammogram. **a** In the outer left breast at posterior depth on the craniocaudal (CC) and mediolateral oblique (MLO) views, there is a subtle architectural distortion. **b**, **c** Diagnostic ultrasound (US) of the left breast, 3:30 position, 3 cm from the nipple shows a 2.8 x 1.5 cm irregular hypoechoic mass with indistinct margins and posterior shadowing; this mass corresponds to the mammographic architectural distortion. **c** There is no appreciable blood flow within the mass on Doppler US. **d**, **e** Dynamic axial T1-weighted post-contrast subtracted MRI images show-delayed maximum enhancement of the biopsy-proven ILC in the outer left breast at posterior depth. There is also enhancement of the underlying pectoralis musculature, consistent with direct tumor invasion. **f** Kinetics color map overlay demonstrates a Type 1 persistent enhancement kinetic profile

**Fig. 3:**
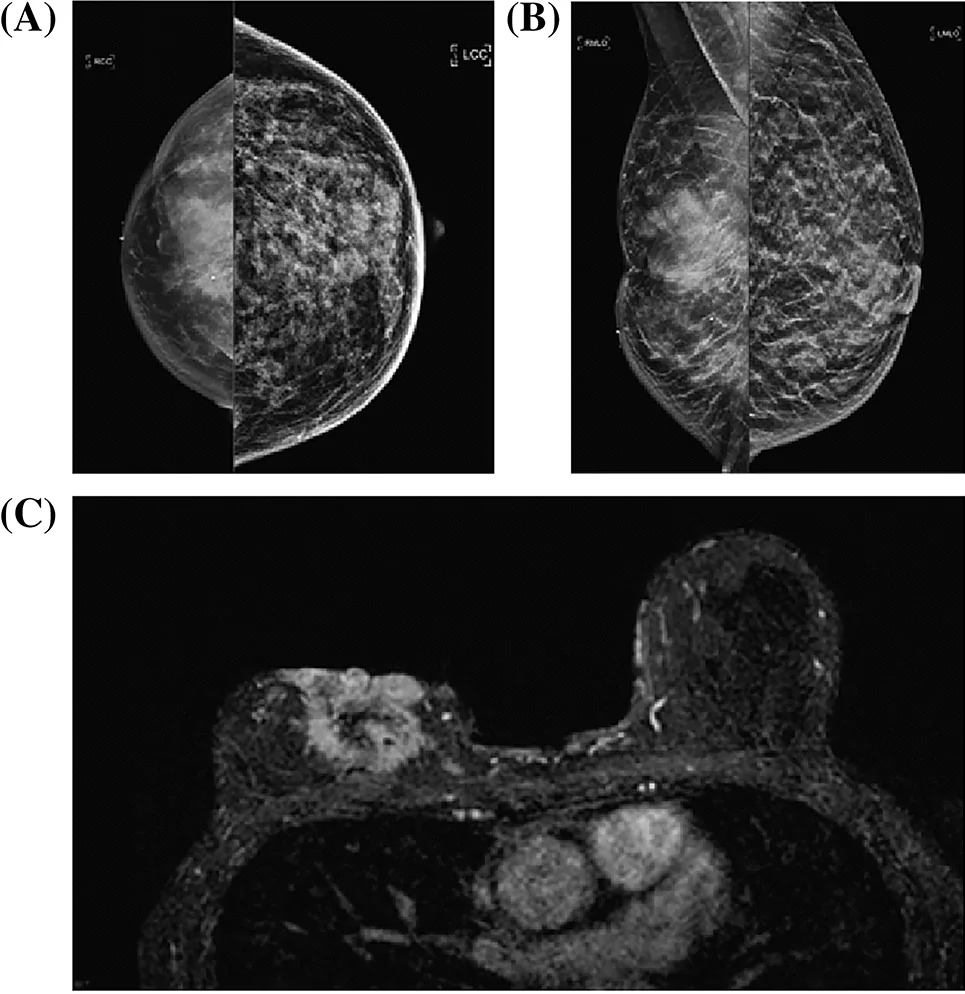
A 63 year-old female presenting with right breast hardness. **a**, **b** Bilateral diagnostic mammogram with craniocaudal (CC) and mediolateral oblique (MLO) views demonstrates asymmetric shrinking of the right breast with nipple retraction, global asymmetry, and diffuse skin thickening. **c** Axial T1 weighted post-contrast breast magnetic resonance imaging (MRI) with subtraction demonstrates clearer extent of multicentric disease, nipple areolar complex and skin involvement, and asymmetric right breast shrinkage

Mammography, ultrasound, and magnetic resonance imaging (MRI) are each important for detecting and evaluating ILC on imaging. Mammography is the least sensitive imaging modality with a reported range of 57–81%, which is decreased in dense breasts.^4–9^ Because the mammographic findings of ILC are often subtle, the false-negative rate for ILC (19–66%) on mammograms is higher compared with invasive ductal carcinoma (19%).^5,10^ The most common mammographic presentation of ILC is a low or equal density mass (68%), typically with spiculated or ill-defined margins (Fig. 1).^11^ Circumscribed oval or round masses are much less common, reported in only 1–3% of cases.^4,6,8^ Architectural distortion is the second most common imaging manifestation of ILC (Fig. 2a), seen in 10–34% of cases.^4–6,8,12,13^ Tomosynthesis increases detection rate of architectural distortion from 0.27 to 0.55 per 1000 cases.^14^ Calcifications are rare and seen much less often compared with invasive ductal carcinoma (IDC).^8^ More advanced cases of ILC may present as an asymmetric “shrinking breast” with possible nipple inversion/retraction and skin thickening (Fig. 3ab).

Contrast-enhanced digital mammography (CEM) is an increasingly utilized breast imaging modality, which has demonstrated promising results for ILC detection and assessment. A multicenter retrospective study found that using CEM instead of MRI for preoperative staging of ILC yielded comparable assessment of disease extent and detection of multifocal and contralateral disease.^15^ Overall, for patients with mild to moderately dense breasts, CEM is reliable for accurate diagnosis and extent of disease assessment.^16^

Ultrasound is more sensitive for ILC detection compared with mammography with an overall sensitivity of 68–98%.^7,9,17^ Most commonly, it presents as a hypoechoic irregular mass with posterior shadowing (Fig. 2bc). Some cases may present as a focal shadowing area without a discrete mass and 10% are sonographically occult.^18^ Similar to mammography, ILC rarely presents as a circumscribed mass. Ultrasound is better at identifying multicentricity and multifocality while more accurately depicting the size of the mass compared with mammography.^11^

Magnetic resonance imaging has the highest sensitivity (95%) of all breast imaging modalities and is superior to ultrasound and mammography for depicting multifocal or bilateral disease.^17,19^ It is the most accurate at assessing tumor extent and size. Staging breast MRI is clinically useful, affecting clinical management in 50% of patients with ILC and changing surgical management in 28% of cases.^20,21^ The American College of Radiology does not specifically recommend routine MRI surveillance based on lobular histology alone in patients with a personal history of breast cancer. However, annual breast MRI is recommended for patients with a mammographically occult primary breast cancer, which is relevant to ILC given its higher false-negative rate on mammography.^22,23^ The most common appearance of ILC is a solitary irregular mass with spiculated or irregular margins; nonmass enhancement is the second most common presentation.^11^ Invasive lobular carcinoma has a tendency to demonstrate delayed maximum enhancement (Fig. 2de), and delayed-phase washout on kinetic analysis is less common in ILC compared with carcinoma of no special type (NST) (Fig. 2f).^21^

### Neoadjuvant Systemic Therapy in ILC

Invasive lobular carcinoma is known to have lower response rates to neoadjuvant chemotherapy, as demonstrated by lower likelihood of pathologic complete response (pCR). In a meta-analysis of 7,596 patients with ILC and 79,708 patients with IDC, the pooled pCR rate for ILC was 7.4% compared with 22.1% for IDC.^24^ Although this is likely attributable to higher rates of well-differentiated, hormone receptor (HR)-positive, HER2-negative tumors among those with ILC, there is considerable histologic and molecular heterogeneity within ILC.^25^ Increasingly, better appreciation for genomic and transcriptomic features of ILC appears to guide selection for neoadjuvant approaches. Given the high rates of positive margins, mastectomies, and high nodal burden seen in patients with ILC, the ability to decrease tumor burden and assess response to therapy is potentially quite helpful in the management of these patients. Importantly, studies have shown that histology alone is not an independent predictor of pCR.^26–28^

A subset of patients with ILC have tumors that are molecularly high-risk based on genomic assays, such as the 21 gene Recurrence Score (Oncotype RS) or MammaPrint testing.^25,29,30^ Among HR+/HER2− cases, features, such as progesterone receptor negativity and high grade, are associated with high-risk genomic score and improved response to NAC.^31,32^ Recent data from the I-SPY2 trial show that among clinically node-positive patients with HR+/HER2− MammaPrint high risk tumors, nodal pCR after NAC was achieved in nearly one third of cases.^32^ Such data support the idea that proper patient selection can identify those who may benefit from NAC. Current indicators of responsiveness to NAC, such as pCR or the Residual Cancer Burden (RCB) method, may need refinement for those with ILC, as these tumors may have nonconcentric response patterns, leading to “large” residual tumors but low tumor density or cellularity.^33^ More data on the optimal endpoints after neoadjuvant systemic therapy in ILC are needed.

Given the high rates of luminal A biology in ILC, neoadjuvant endocrine therapy (NET) may be more appropriate than NAC for most patients. There are limited data pertaining to NET specifically for ILC, but it has been shown to increase rates of successful BCS in 34-70% of postmenopausal HR+ breast cancer and can downstage the axilla in up to 20% of appropriately selected genomic low risk patients.^34–40^ Longer duration of NET (≥6 months) may be associated with improved surgical outcomes among women with ILC, with continued response reported with NET for up to 12 months, and is a valuable option for patients desiring breast conservation and reduced surgical morbidity.^41,42^

Currently, there are ongoing neoadjuvant trials either enriched for patients with ILC, or specifically for those with ILC. These trials employ both targeted therapies (e.g. neoadjuvant neratinib for those with ERBB2 mutations, which are more common in lobular tumors), or combinations of endocrine therapy with CDK4/6 inhibitors.^43^

### Surgical Management of the Breast in ILC

Both the diffuse growth pattern and difficulties with accurate tumor assessment on imaging lead to challenges with surgical management for patients with ILC.^44^ A major challenge is the high rate of positive margins at breast conserving surgery (BCS), which occurs in 17-59% of patients with ILC.^45–49^ Because of this, rates of second operations and completion mastectomies are higher for those with ILC compared to those with NST).^50^ If clear margins are obtained, however, BCS with radiotherapy appears to offer similar locoregional control as mastectomy, even in patients with large ILC tumors.^51^

Certain surgical techniques have been shown to potentially reduce the risk of positive margins for those with ILC.^52^ First, the routine use of shave margins is strongly associated with lower odds of positive margins.^53^ While routine shave margins have been shown to help reduce positive margins for patients with breast cancer in general, this is even more important in those with high baseline risk of incomplete excision, such as in the case of tumors with lobular histology. Second, incorporation of oncoplastic techniques facilitates resection of larger breast volumes.^53^ This translates to lower odds of positive margins in the setting of lobular tumors. While some have raised concerns about oncoplastic surgery and tissue rearrangement making re-excision more challenging or imprecise should positive margins occur, recent data support the oncologic safety of immediate oncoplastic surgery.^54^ Not only are recurrence rates the same in patients who have immediate oncoplastic surgery versus standard BCS, but positive margin rates are significantly lower in those undergoing immediate oncoplastic surgery.

While some patients with ILC may elect to undergo mastectomy, or have such extensive disease that BCS is technically not feasible, it is important to note that even mastectomy may not reliably clear margins for patients with these diffusely growing tumors. Data suggest that up to 19% of patients with T3 ILC have positive margins at mastectomy.^55^ When positive margins occur after mastectomy, it is not clear whether post-mastectomy radiotherapy versus further excision of soft tissue is the optimal approach. However, some additional local therapy should be employed (radiotherapy, re-excision, or both) to reduce the risk of local recurrence.^55^ Studies have shown that skin-sparing and nipple-sparing mastectomy with immediate reconstruction are not associated with worse outcomes compared to simple or total mastectomy in those with ILC.^56^

Because of the lower sensitivity of imaging tools in the setting of lobular tumors, surgeons must interpret findings with caution and anticipate longer tumor extent than depicted on imaging. The high positive margin rates seen at BCS and even mastectomy in large ILC tumors may support the use of neoadjuvant approaches, not necessarily to facilitate BCS in all cases, but to improve surgical outcomes such as margin clearance for especially large tumors. Further study to improve the surgical approach to the breast in patients with ILC is needed.

### Surgical Management of the Axilla in Invasive Lobular Carcinoma

ILC poses distinct challenges in axillary management. Nodal metastatic disease from ILC may produce only minimal stromal response, a lack of overt morphologic change, and more frequently manifests as isolated tumor cells.^57–59^ As a result, nodal metastasis in ILC may be underestimated pre-operatively, with imaging modalities demonstrating lower sensitivity for detection of nodal disease compared to IDC.^60–62^ Although some studies have demonstrated a higher rate of nodal metastases in ILC compared to IDC,^63^ other data have shown that this phenomenon may be due to the tendency of ILC to present with larger tumor sizes and more multifocality than in IDC; once these primary tumor factors are controlled, the true rate of nodal positivity in ILC may actually be lower than that in IDC when comparing unifocal tumors of the same size.^64,65^ Similarly, although the incidence of non-sentinel node metastasis in ILC is higher than in IDC when sentinel nodes are positive, this residual nodal burden may also be predicted by primary tumor characteristics, with smaller ILCs that meet Z0011 criteria (cT1-2N0, no extra-nodal extension) showing limited non-SLN disease.^66,67^

Considering the difficulty in preoperative estimation of nodal burden in ILC, and the small populations of ILC included in some landmark axillary management trials, surgeons may question whether de-escalation of axillary surgery may be safely applied in patients with ILC. Evidence from recent trials supports the omission of sentinel lymph node biopsy (SLNB) and axillary lymph node dissection (ALND) in carefully selected patients with ILC. The SOUND and INSEMA trials demonstrated equivalent oncologic outcomes (and extraordinarily low rates of axillary recurrence) in patients with early-stage breast cancer and negative axillary ultrasound, whether or not SLNB was performed.^68,69^ The INSEMA trial was large enough to include a subgroup analysis for ILC, similarly showing equivalent outcomes to IDC. However, this subgroup analysis was underpowered to find a difference and thus omission of SLNB in ILC per SOUND and INSEMA remains controversial. As ILC is known to have higher risk of late recurrence, longer follow up time is likely needed to determine the true impact of omission of axillary surgery in this patient population. A subset analysis of the SINODAR-ONE trial found that lobular histology was an independent predictor of high nodal burden (N2 disease). Current guidelines from the American Society of Clinical Oncology do not endorse omission of SLNB in those with ILC.^70,71^ Importantly, the inclusion criteria of these trials must be carefully considered when deliberating whether omission of SLNB may be applied for ILC as the size of most primary tumors was <2 cm, and the tumors were overwhelmingly hormone receptor positive.

Data from the ACOSOG Z0011 and SENOMAC trials confirmed that ALND can be safely omitted in breast cancer patients with limited nodal involvement on SLNB.^72,73^ The SENOMAC trial included a subgroup analysis for ILC and demonstrated equivalent outcomes in the SLNB only and ALND groups. Moreover, retrospective SEER data indicate no survival advantage with ALND over SLNB alone in Z0011-eligible ILC patients,^74^ supporting the safety of axillary de-escalation approaches in appropriately selected ILC cases. Careful attention to pre-operative imaging, physical examination, use of radiotherapy, ability to tolerate adjuvant systemic therapy, and patient age are all essential to identify appropriate candidates.

While neoadjuvant chemotherapy (NAC) and neoadjuvant endocrine therapy (NET) offer potential downstaging of nodal disease, ILC demonstrates low rates of nodal pathologic complete response (pCR) following such therapies, likely related to the predominantly hormone receptor positive nature of ILC.^75,76^ In fact, neoadjuvant systemic therapy may inadvertently increase the likelihood of ALND in ILC due to persistent nodal involvement, based on current management paradigms that generally require ALND for any residual disease after NAC which may not have been indicated with surgery prior to systemic therapy. Consequently, upfront surgery remains a strategically favorable option for many patients with hormone receptor positive ILC.

Taken together, these findings reinforce the applicability of contemporary axillary management paradigms to ILC while highlighting the need for nuanced clinical judgment, particularly in tailoring decisions on SLNB and ALND based on tumor characteristics, imaging findings, and eligibility under established trial criteria.

### Genetic Testing Concerns in ILC

A pathogenic or likely pathogenic (PLP) variant in the *CDH1* gene carries increased risk of developing hereditary lobular breast cancer (HLBC), a familial form of ILC, and has nuanced management. The classic characteristic of ILC is a discohesive morphology that is thought to be attributed to loss of E-cadherin function.^77^ E-cadherin is a transmembrane glycoprotein that is transcribed from the *CDH1* gene located on chromosome 16q22.1. It is an integral part of the adherens junctions between epithelial cells, acting in cell-to-cell adhesion and signaling. It is vital in maintaining tissue structural stability and dynamic cell movements. Loss of E-cadherin function is associated with cell motility, invasion, and epithelial-mesenchymal transition.^78–80^

In addition to developing ILC, *CDH1* variant carriers are linked to diffuse-type gastric cancer. In the gastric mucosa, loss of function mutations in *CDH1* (the gene encoding for E-cadherin) result in gastric signet ring cell (SRC, T1a invasive disease) formation. While it has been established that while nearly all (95-100%) of PLP *CDH1* variant carriers harbor dormant SRC in their gastric mucosa, the estimated lifetime risk of a PLP *CDH1* variant carrier developing clinically relevant diffuse gastric cancer is only 3-23%.^81,82^ Comparatively, the lifetime risk of developing breast cancer is much higher between 25 and 62%.^81^ Currently, there are no known *CDH1* variants in which the predominant presentation is isolated familial ILC, but rather all variants carry the risk of both ILC and diffuse-type gastric cancer.^83,84^

For any new ILC diagnosis, in addition to criteria for genetic testing set forth by the National Comprehensive Cancer Network (NCCN), 2020 American Society of Breast Surgeons consensus guidelines recommend genetic testing on any family with two or more cases of ILC less than 50 years, any individual with bilateral ILC less than 70 years, any family with a case of diffuse gastric cancer and ILC, and any family with two or more cases of gastric cancer.^82^ In areas with limited access to genetic testing, strong family history of diffuse gastric cancer in an individual with ILC should warrant referral to a center with access to testing, when such a center is available. If a germline *CDH1* variant is identified, breast cancer surveillance should start at 30 years with yearly MRI alternating with yearly mammogram at 40 years while bilateral risk-reducing mastectomy may alternatively be considered. Consensus guidelines also recommend prophylactic gastrectomy as early as 20 years while annual surveillance endoscopy is an option for those declining or delaying gastrectomy.^82^

In patients with a germline PLP *CDH1* variant diagnosed with ILC, current guidelines suggest that standard breast conserving therapy can be appropriate treatment when negative margins are attainable and radiation therapy is not contraindicated. However, the lifetime risk of developing an in-breast tumor recurrence or contralateral breast cancer is not known in this population and mastectomy with contralateral prophylactic mastectomy is a reasonable alternative particularly in the premenopausal female patient.

When considering treatment of ILC in a patient with a known PLP *CDH1* variant and personal history of gastric cancer it is imperative to understand the extent of disease in the stomach and anticipated prognosis. Specifically, since we know that all PLP *CDH1* variant carriers have gastric mucosal lesions (SRC) this discovery on endoscopy should not affect treatment decisions that pertain to breast ILC because these occult gastric lesions do not compromise the patients’ lifespan. Conversely, if a patient has a personal history of advanced gastric cancer (invasion into or beyond the gastric muscularis mucosa) then this should be considered in conjunction with appropriate and updated staging work-up as a patients’ lifespan can be severely compromised.

While *CDH1* is a cornerstone for ILC, there are many other germline variants that predispose individuals to developing breast cancer and patients with ILC should undergo genetic testing per NCCN guidelines. Indeed, alterations in BRCA2 have been reported as the most common pathogenic variant in those with ILC.^85^ Pathogenic variants in *BRCA1/2* increase the lifetime risk of breast cancer up to 80% and are known for predilection of a triple negative receptor status.^86^ *CHEK2* is associated with ER-positive breast cancers and is thought to increase lifetime risk up to 40%.^87^ Lynch syndrome, also called hereditary nonpolyposis colon cancer, is associated with PLP variants in mismatch repair genes (*MSH2, MLH2, MSH6,* and *PMS2*) as well as the epithelial cell adhesion molecule gene (*EPCAM*). These variants carry increased risk of developing colon, endometrial, ovarian, stomach cancer and some studies suggest that variants in *MSH6* and *PMS2* may increase risk of breast cancer up to 36%. Less studied genes *PALB2* and *ATM* are also thought to increase risk up to 32% and 25%, respectively, while *BARD1, RAD51C, and RAD51D* have not shown any increased lifetime risk of breast cancer development, though they have been moderately associated with ER-negative breast cancers.^88^

## Discussion

The multidisciplinary management of ILC is nuanced and special considerations are essential for optimizing care (Table 1). Because of the discohesive growth pattern of ILC, imaging with MRI can improve the accuracy of preoperative staging. Surgeons should account for the limitations of imaging with respect to margin resection in lumpectomy and application of data on axillary management. Use of cavity shave margins and oncoplastic lumpectomy are two techniques that can be applied to reduce the risk of positive margins. When considering the application of trials guiding omission of ALND and SLNB in surgery for ILC, surgeons can rely on several studies that have included sub-analyses of patients with ILC confirming that the trial results are the same in ILC as in IDC.

**Table 1:** Summary of management considerations specific to ILC

Considerations in management of invasive lobular carcinoma (ILC)
*Imaging in ILC*
Sensitivity of mammography is lower with higher rate of false negatives. Both MRI and ultrasound have higher sensitivity for ILC detection than mammography. MRI is the most accurate imaging modality in assessing tumor extent, size and multifocality. Use of preoperative MRI should be considered.
*Surgical management of the breast*
Positive margin rates after surgical resection of ILC is higher than IDC. Strategies to reduce positive margin rates after breast conserving surgery for ILC include preoperative MRI and cavity shave margins. Oncoplastic surgery in the setting of ILC has been shown to be ontologically safe and can be utilized to reduce the need for mastectomy.
*Surgical management of the axilla*
Small populations of ILC have been included in landmark axillary management trials (e.g., ACOSOG Z0011, SENOMAC); however, subgroup analyses and retrospective data have consistently supported safety of axillary de-escalation in ILC patients. De-escalation of axillary surgery per SOUND & INSEMA in patients with ILC remains controversial due to underpowered subgroup analyses. Low nodal pathologic complete response rates after neoadjuvant chemotherapy has been observed in ILC; neoadjuvant therapy solely to downstage the axilla vs. surgery first requires an individualized and multidisciplinary discussion.
*Neoadjuvant therapy*
Molecular tumor subtype (e.g., hormone receptor [HR] and HER2 status, genomic risk profile) should guide neoadjuvant therapy decision making, not histologic subtype (e.g., ILC vs. ID). Neoadjuvant endocrine therapy may improve breast conserving surgery eligibility in HR+ HER2− ILC and avoid systemic toxicity of chemotherapy.
*Genetics*
Patients with ILC should undergo genetic testing per NCCN Guidelines. Germline pathogenic or likely pathogenic (P/LP) variants in *CDH1* present in patients with ILC more frequently than IDC. Individuals with P/LP germline variants in CDH1 require high-risk gastric cancer screening as well as breast cancer screening, if identified.

Response to systemic therapy in ILC is consistent with its biology which is most commonly luminal A type tumor that is strongly HR+. Neoadjuvant therapy with NAC less likely to lead to pCR in most ILC which is HR+ and the data support management of ILC with neoadjuvant therapy based on molecular subtype, determined by expression of ER, PR and HER2, rather than tumor histology. The use of NET is promising in ILC that is HR+ for improving breast conservation rates. There is a genetic link for some LBC to germline mutations in the *CDH1* gene. It has been proposed that all patients with ILC undergo genetic testing because of this link. Those ordering genetic testing for patients with ILC should include testing for *CDH1* mutations.

Beyond the topics covered in the present review, additional management decisions are unique to ILC remain. Histologically, compared to IDC, ILC is more often associated with both lobular carcinoma in situ (LCIS) and atypical hyperplasia (ALH), collectively referred to as lobular neoplasia (LN).^89^ The management of surgical margins after breast conserving surgery in the presence of LCIS and ALH remains controversial.^90^ Expert consensus recommends that the presence of ALH or classic LCIS at or near a surgical margin should not influence the decision for re-excision, however no clear guidelines or consensus exists on the management of either pleomorphic or florid LCIS at or near the surgical margin.^91^ A recent large, single institution study of 511 patients with stage 0-III breast cancer associated with non-classic LCIS demonstrated that distance of non-classic LCIS from the surgical margin was not associated with risk of local recurrence with a median follow up of 3.4 years (IQR 2.0-5.9), suggesting that non-classic LCIS should not drive the decision to re-excise although longer follow up is likely needed.^92^ Additionally, the presence of LN associated with either invasive breast cancer or DCIS has been associated with an increased risk of a contralateral breast cancer.^89,93^ Braasch et al identifying a risk ratio of 3.3 [95% CI 1.5-7.3] for a future contralateral breast cancer in a cohort of 301 patients with invasive breast cancer, predominantly lobular histology, associated with LN compared to the risk of contralateral breast cancer in 1507 patients with invasive breast cancer without LN with a mean follow up of <3 years.^89^ Similarly, Miller et al. from Memorial Sloan Kettering identified an increased risk of contralateral breast cancer with a hazard ratio of 2.06 (95% CI 1.36-3.11) in those patients with DCIS associated with LCIS compared to patients with DCIS alone with a median follow up of 7.2 years (range 0-17).^93^ These studies raise the question whether counseling on future screening practices, including addition of MRI, should be routinely recommend or if counseling regarding contralateral prophylactic mastectomy should be tailored for patients with LN associated with their invasive disease; however, currently no formal guidelines exist and additional prospective, multi-institutional, long term follow up is needed to provide generalizable data that is necessary to guide the development of formal guidelines.

While the present study discusses decision making around neoadjuvant therapy in ILC, decisions around adjuvant therapy for ILC are also nuanced. As most ILC is positive by IHC for ER and/or PR and is HER2 negative, validated genomic assays including Oncotype DX and Mammaprint are commonly used to guide adjuvant therapy decision making. However, ILC patients were under-represented in the MINDACT, TAILORx, and RxPONDER trials validating the use of these genomic assays for recurrence risk prediction and chemotherapy benefit.^94–96^ Although follow up studies have been completed to validate the accuracy of these genomic assays in ILC patients specifically, it has been shown that a higher proportion of ILC patients have low risk genomic scores.^25,97,98^ As the genomic landscape for ILC has been shown to differ from IDC, additional groups have worked to develop ILC specific genomic assays, although none are currently clinically available and additional external validation is likely necessary prior to clinical use.^99^ Beyond use of genomic assays, for novel breast cancer therapies, a recent systematic review identified that of phase III/IV breast cancer clinical trials, inclusion of ILC was only documented in 13/93 manuscripts and the proportion of ILC patients included varied from 2-26%.^100^ As treatment options continue to advance and expand, given the often differential response to therapy and other unique differences observed in ILC,^101^ inclusion of patients with ILC and planned sub-cohort analyses are crucial to continuing to advance our understanding of optimal treatment for ILC.

Pattern and timing of recurrence has also been shown to be unique to patients with ILC. In combined analysis of 15 clinical trials from the International Breast Cancer Study Group that included 12,260 breast cancer patients including 767 with ILC, patients with ILC had better DFS and OS with <6 year follow-up, but worse DFS and OS with follow-up beyond 6 and 10 years, suggesting that recurrences tend to occur later in patients with ILC.^101^ Beyond timing of recurrence, pattern of recurrence also differs. Compared to IDC, ILC more frequently metastasizes to the bone, ovaries and gastrointestinal tract and is less likely to recur locally or metastasize to the lungs or liver.^101–103^ Currently, NCCN guidelines do not differentiate recommendations for surveillance between patients with ILC versus IDC; however clinicians should be aware of the differential timing and potential location of metastases in ILC when caring for patients undergoing surveillance.

Limitations exist in the present study. This is not a formal systematic review, but is rather an expert panel of many issues related to the management of ILC. This review is therefore certainly not exhaustive of all studies that have previously been published nor all nuances that exist regarding management of patients with ILC due to the nature of the methodology. Rather it is meant to highlight important topics noted by the expert panel.

## Conclusion

As the second most common histologic subtype of breast cancer, ILC poses unique and differential management of which clinicians caring for breast cancer patients must be aware. Pre-operative imaging is often less accurate with underestimation of disease extent and higher rates of positive margins after breast surgery have been reported, although this may be improved by obtaining a pre-operative breast MRI. Invasive lobular carcinoma is often underrepresented in clinical trials regarding both systemic therapy and axillary management, including recent trials focused on SLNB omission following normal axillary ultrasound. Neoadjuvant systemic therapy decisions should therefore be based on molecular subtype, rather than histologic subtype. Additionally, germline genetic testing when indicated should include *CDH1*. Awareness and consideration of these nuances around the management of ILC can help clinicians provide optimal, evidenced-based care to their patients.
